# Bis{1-[(1*H*-benzotriazol-1-yl)meth­yl]-2-methyl-1*H*-imdazole-κ*N* ^3^}dichlorido­zinc

**DOI:** 10.1107/S1600536812022313

**Published:** 2012-05-26

**Authors:** Haiyan Yang, Yinghua Li

**Affiliations:** a School of Materials and Chemical Engineering, Zhongyuan University of Technology, Zhengzhou 450007, People’s Republic of China

## Abstract

In the mononuclear title compound, [ZnCl_2_(C_11_H_11_N_5_)_2_], the Zn^II^ atom is coordinated by two Cl atoms and two imidazole N atoms in a distorted tetra­hedral geometry. Adjacent complex mol­ecules are stacked through aromatic π–π inter­actions; the closest distance between adjacent aromatic rings is 3.598 (2) Å.

## Related literature

For an introduction to metal-organic networks, see: Chen *et al.* (2001 ▶). For general background to complexes constructed from *N*-heterocyclic ligands, see: Yang *et al.* (2009 ▶); Meng *et al.* (2009 ▶); Zhao *et al.* (2012*a* ▶,*b* ▶). For π–π inter­actions, see: Janiak *et al.* (2000 ▶).
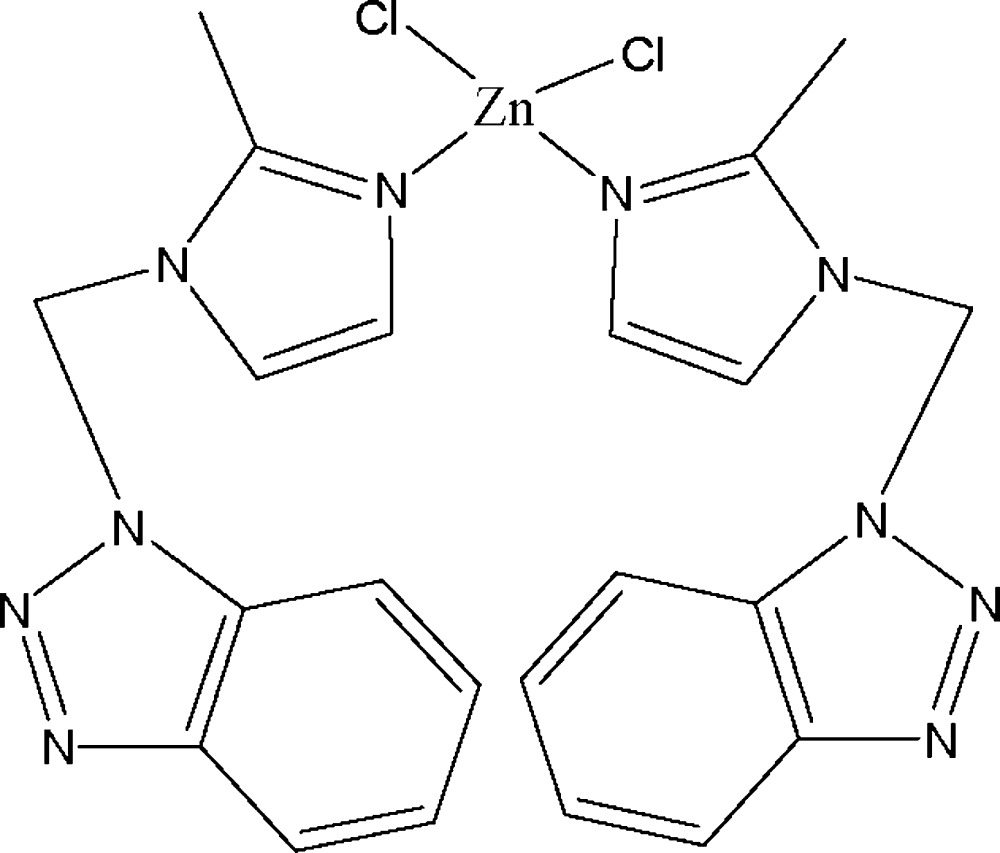


## Experimental

### 

#### Crystal data

[ZnCl_2_(C_11_H_11_N_5_)_2_]
*M* *_r_* = 562.79
Triclinic, 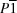

*a* = 8.1625 (16) Å
*b* = 12.692 (3) Å
*c* = 13.290 (3) Å
α = 65.52 (3)°
β = 79.47 (3)°
γ = 84.02 (3)°
*V* = 1231.3 (6) Å^3^
*Z* = 2
Mo *K*α radiation
μ = 1.25 mm^−1^
*T* = 295 K
0.21 × 0.20 × 0.18 mm

#### Data collection

Rigaku Saturn CCD diffractometer
Absorption correction: numerical (*REQAB*; Jacobson, 1998 ▶) *T*_min_ = 0.780, *T*_max_ = 0.807
9045 measured reflections
4291 independent reflections
3870 reflections with *I* > 2σ(*I*)
*R*_int_ = 0.020

#### Refinement

*R*[*F* ^2^ > 2σ(*F* ^2^)] = 0.033
*wR*(*F* ^2^) = 0.079
*S* = 1.02
4291 reflections
318 parameters
H-atom parameters constrained
Δρ_max_ = 0.25 e Å^−3^
Δρ_min_ = −0.21 e Å^−3^

### 

Data collection: *CrystalClear* (Rigaku/MSC, 2006 ▶); cell refinement: *CrystalClear*; data reduction: *CrystalClear*; program(s) used to solve structure: *SHELXS97* (Sheldrick, 2008 ▶); program(s) used to refine structure: *SHELXL97* (Sheldrick, 2008 ▶); molecular graphics: *SHELXTL* (Sheldrick, 2008 ▶); software used to prepare material for publication: *SHELXTL*.

## Supplementary Material

Crystal structure: contains datablock(s) global, I. DOI: 10.1107/S1600536812022313/rk2337sup1.cif

Structure factors: contains datablock(s) I. DOI: 10.1107/S1600536812022313/rk2337Isup2.hkl

Additional supplementary materials:  crystallographic information; 3D view; checkCIF report

## Figures and Tables

**Fig. 1.**
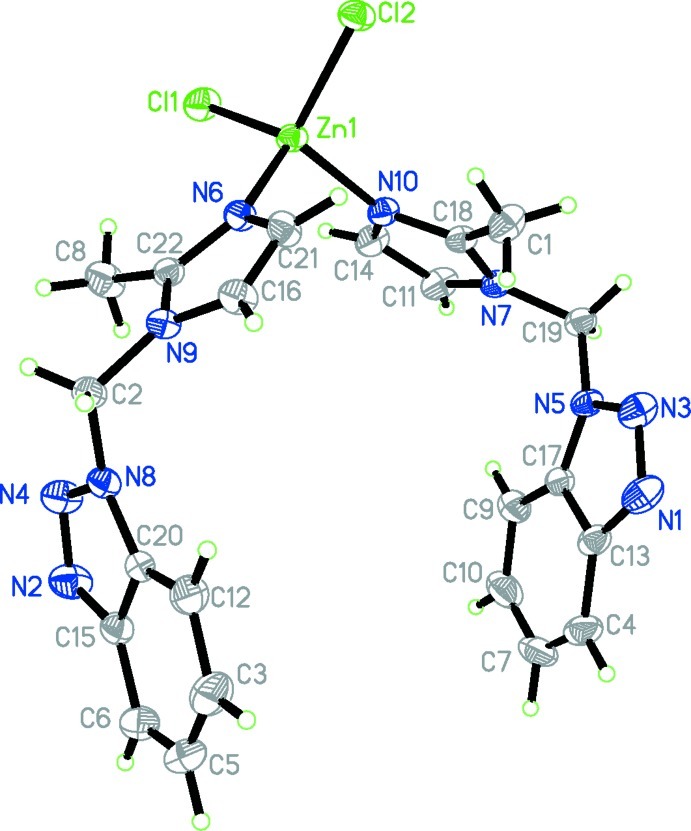
View of the title nolecule, showing the atom labeling scheme. Displacement ellipsoids are drawn at the 30% probability level. H atoms are presented as a small spheres of arbitrary radius.

**Fig. 2.**
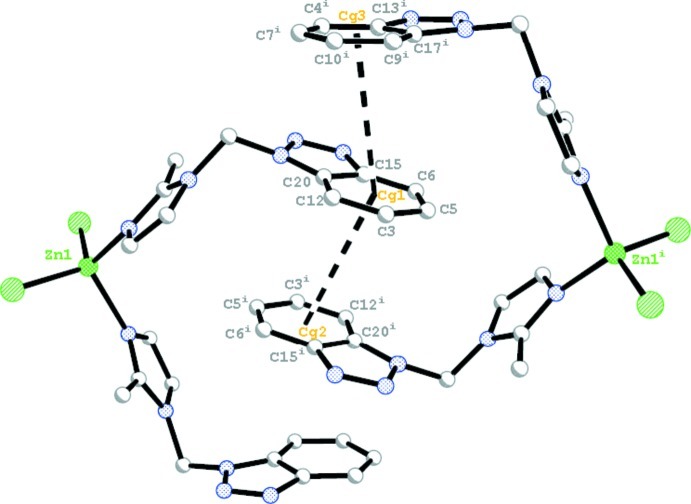
The π–π-stacking interactions between the two adjacent mononuclear structure units. Symmetry code: (i) -x, -y, -z.

**Fig. 3.**
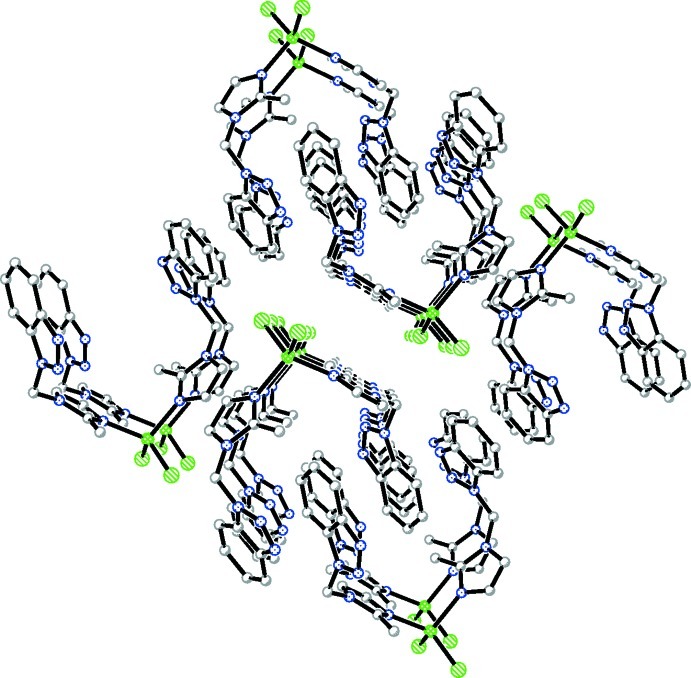
View of the crystal packing along the a axis.

## supplementary crystallographic information

### Comment

In recent years, metal-organic networks have been widely developed because of the charming structure topologies and applications in various areas (Chen *et al.*, 2001). The flexible multidentate *N*-heterocyclic ligands are good candidates for constructing metal-organic networks due to their diverse coordination modes (Yang *et al.*, 2009; Meng *et al.*, 2009; Zhao *et al.*, 2012*a*; Zhao *et al.*, 2012*b*). In this work, we selected a new *N*-heterocyclic compound 1-[(benzotriazol-1-yl)methyl]-1-*H*-1,3-(2-methyl-imdazol) as ligand, generating a new coordination compound, Zn(C_11_H_11_N_5_)2Cl_2_, (I), which is reported here. In the I, the Zn atom is four-coordinated by two N atoms from two ligands and two Cl atoms in a distorted tetrahedral geometry with the dihedral angle of 84.1 (2)° between the N6/Zn1/N10 and Cl1/Zn1/Cl2 planes (Fig. 1). In the crystal structure, the two adjacent mononuclear structure units are stacked through the aromatic π–π-stacking interactions (the closest distance between adjacent aromatic rings is 3.598 (2)Å), forming a quasi-dinuclear structure (Fig. 2), and these quasi-dinuclear structure units are further linked by intermolecular interactions to form a three-dimensional supramolecular network (Fig. 3).

### Experimental

A methanol solution (5 ml) of 1-[(benzotriazol-1-yl)methyl]-1-*H*-1,3-(2-methyl-imdazol) (21.3 mg, 0.1 mmol) was added dropwise into a methanol–aqueous solution (3 ml) of ZnCl_2_ (0.05 mmol, 6.8 mg). The precipitate was filtered and the resulting solution was left at room temperature. After three days, good quality colourless crystals were obtained from the filtrate and dried in air.

### Refinement

H atoms were generated geometrically, with C—H = 0.96, 0.97 and 0.93Å for methyl, methylene and aromatic H, respectively, and constrained to ride their parent atoms with *U*_iso_(H) = 1.5*U*_eq_(C) for methyl H and *U*_iso_(H) = 1.2*U*_eq_(C) for other H atoms.

### Crystal data

**Table:**

[ZnCl_2_(C_11_H_11_N_5_)_2_]	*Z* = 2
*M**_r_* = 562.79	*F*(000) = 576
Triclinic, *P*1	*D*_x_ = 1.518 Mg m^−^^3^
Hall symbol: -P 1	Mo *K*α radiation, λ = 0.71073 Å
*a* = 8.1625 (16) Å	Cell parameters from 3518 reflections
*b* = 12.692 (3) Å	θ = 2.5–26.0°
*c* = 13.290 (3) Å	µ = 1.25 mm^−^^1^
α = 65.52 (3)°	*T* = 295 K
β = 79.47 (3)°	Block, colourless
γ = 84.02 (3)°	0.21 × 0.20 × 0.18 mm
*V* = 1231.3 (6) Å^3^	

### Data collection

**Table:**

Rigaku Saturn CCD diffractometer	4291 independent reflections
Radiation source: fine-focus sealed tube	3870 reflections with *I* > 2σ(*I*)
Graphite monochromator	*R*_int_ = 0.020
Detector resolution: 28.5714 pixels mm^-1^	θ_max_ = 25.0°, θ_min_ = 2.5°
ω scans	*h* = −9→9
Absorption correction: numerical (*REQAB*; Jacobson, 1998)	*k* = −15→15
*T*_min_ = 0.780, *T*_max_ = 0.807	*l* = −15→15
9045 measured reflections	

### Refinement

**Table:**

Refinement on *F*^2^	Secondary atom site location: difference Fourier map
Least-squares matrix: full	Hydrogen site location: inferred from neighbouring sites
*R*[*F*^2^ > 2σ(*F*^2^)] = 0.033	H-atom parameters constrained
*wR*(*F*^2^) = 0.079	*w* = 1/[σ^2^(*F*_o_^2^) + (0.0395*P*)^2^ + 0.550*P*] where *P* = (*F*_o_^2^ + 2*F*_c_^2^)/3
*S* = 1.02	(Δ/σ)_max_ = 0.001
4291 reflections	Δρ_max_ = 0.25 e Å^−^^3^
318 parameters	Δρ_min_ = −0.21 e Å^−^^3^
0 restraints	Extinction correction: *SHELXL97* (Sheldrick, 2008)
Primary atom site location: structure-invariant direct methods	Extinction coefficient: 0.0016 (4)

### Special details

**Table:**

Geometry. All s.u.'s (except the s.u. in the dihedral angle between two l.s. planes) are estimated using the full covariance matrix. The cell s.u.'s are taken into account individually in the estimation of s.u.'s in distances, angles and torsion angles; correlations between s.u.'s in cell parameters are only used when they are defined by crystal symmetry. An approximate (isotropic) treatment of cell s.u.'s is used for estimating s.u.'s involving l.s. planes.
Refinement. Refinement of *F*^2^ against ALL reflections. The weighted *R*-factor *wR* and goodness of fit *S* are based on *F*^2^, conventional *R*-factors *R* are based on *F*, with *F* set to zero for negative *F*^2^. The threshold expression of *F*^2^ > σ(*F*^2^) is used only for calculating *R*-factors(gt) *etc*. and is not relevant to the choice of reflections for refinement. *R*-factors based on *F*^2^ are statistically about twice as large as those based on *F*, and *R*-factors based on ALL data will be even larger.

### Fractional atomic coordinates and isotropic or equivalent isotropic displacement parameters (Å^2^)

**Table:**

	*x*	*y*	*z*	*U*_iso_*/*U*_eq_	
Zn1	0.26096 (3)	0.74531 (2)	0.46846 (2)	0.03355 (10)	
C1	−0.0640 (4)	0.7685 (2)	0.6732 (2)	0.0512 (7)	
H1A	−0.0589	0.7233	0.6299	0.077*	
H1B	−0.1713	0.8072	0.6750	0.077*	
H1C	−0.0469	0.7186	0.7481	0.077*	
C2	0.6003 (3)	0.3826 (2)	0.7613 (2)	0.0409 (6)	
H2A	0.5611	0.3045	0.8044	0.049*	
H2B	0.6908	0.3796	0.7039	0.049*	
C3	0.5257 (4)	0.2904 (3)	1.1385 (2)	0.0641 (8)	
H3	0.4495	0.2423	1.1963	0.077*	
C4	0.1027 (4)	0.8220 (3)	1.1020 (2)	0.0606 (9)	
H4	0.0662	0.7702	1.1748	0.073*	
C5	0.6498 (4)	0.3382 (3)	1.1651 (2)	0.0613 (8)	
H5	0.6543	0.3204	1.2399	0.074*	
C6	0.7635 (4)	0.4097 (3)	1.0851 (2)	0.0531 (7)	
H6	0.8446	0.4423	1.1032	0.064*	
C7	0.2358 (5)	0.8891 (3)	1.0776 (3)	0.0692 (10)	
H7	0.2905	0.8839	1.1351	0.083*	
C8	0.6422 (3)	0.6102 (3)	0.5543 (2)	0.0539 (7)	
H8A	0.6301	0.6618	0.4789	0.081*	
H8B	0.7257	0.5513	0.5533	0.081*	
H8C	0.6753	0.6530	0.5919	0.081*	
C9	0.2172 (3)	0.9801 (2)	0.8796 (2)	0.0505 (7)	
H9	0.2551	1.0317	0.8070	0.061*	
C10	0.2928 (4)	0.9661 (3)	0.9675 (3)	0.0662 (9)	
H10	0.3859	1.0092	0.9543	0.079*	
C11	0.2104 (3)	1.0085 (2)	0.5854 (2)	0.0475 (6)	
H11	0.2452	1.0755	0.5865	0.057*	
C12	0.5123 (3)	0.3121 (2)	1.0295 (2)	0.0499 (7)	
H12	0.4294	0.2806	1.0118	0.060*	
C13	0.0228 (3)	0.8343 (2)	1.0126 (2)	0.0423 (6)	
C14	0.2853 (3)	0.9530 (2)	0.5215 (2)	0.0449 (6)	
H14	0.3822	0.9758	0.4698	0.054*	
C15	0.7536 (3)	0.4324 (2)	0.9738 (2)	0.0399 (6)	
C16	0.2988 (3)	0.4307 (2)	0.7382 (2)	0.0424 (6)	
H16	0.2515	0.3673	0.7993	0.051*	
C17	0.0800 (3)	0.9127 (2)	0.90483 (19)	0.0361 (5)	
C18	0.0675 (3)	0.85568 (19)	0.62160 (18)	0.0337 (5)	
C19	−0.0489 (3)	0.9778 (2)	0.72795 (19)	0.0390 (6)	
H19A	−0.0363	1.0582	0.7134	0.047*	
H19B	−0.1604	0.9702	0.7164	0.047*	
C20	0.6307 (3)	0.38376 (19)	0.94853 (19)	0.0336 (5)	
C21	0.2188 (3)	0.5156 (2)	0.6632 (2)	0.0413 (6)	
H21	0.1043	0.5207	0.6632	0.050*	
C22	0.4817 (3)	0.5555 (2)	0.61400 (18)	0.0337 (5)	
Cl1	0.45953 (8)	0.83942 (6)	0.32652 (5)	0.04840 (17)	
Cl2	0.03843 (8)	0.70774 (5)	0.41322 (5)	0.04285 (16)	
N1	−0.1179 (3)	0.78472 (19)	1.01042 (18)	0.0547 (6)	
N2	0.8546 (3)	0.4955 (2)	0.87684 (19)	0.0533 (6)	
N3	−0.1494 (3)	0.82872 (19)	0.90881 (18)	0.0495 (6)	
N4	0.8026 (3)	0.48747 (19)	0.79389 (18)	0.0489 (6)	
N5	−0.0311 (2)	0.90653 (16)	0.84270 (15)	0.0354 (4)	
N6	0.3321 (2)	0.59458 (16)	0.58550 (15)	0.0348 (4)	
N7	0.0725 (2)	0.94689 (16)	0.64867 (15)	0.0350 (4)	
N8	0.6644 (2)	0.42128 (16)	0.83454 (16)	0.0364 (4)	
N9	0.4650 (2)	0.45578 (16)	0.70724 (15)	0.0347 (4)	
N10	0.1957 (2)	0.85662 (16)	0.54477 (15)	0.0355 (4)	

### Atomic displacement parameters (Å^2^)

**Table:**

	*U*^11^	*U*^22^	*U*^33^	*U*^12^	*U*^13^	*U*^23^
Zn1	0.03514 (17)	0.03640 (16)	0.02612 (15)	−0.00623 (11)	−0.00370 (11)	−0.00895 (11)
C1	0.0563 (18)	0.0530 (16)	0.0458 (15)	−0.0214 (13)	0.0089 (13)	−0.0238 (13)
C2	0.0439 (15)	0.0411 (13)	0.0426 (14)	0.0049 (11)	−0.0164 (12)	−0.0192 (11)
C3	0.059 (2)	0.077 (2)	0.0445 (17)	−0.0119 (16)	0.0098 (15)	−0.0178 (16)
C4	0.085 (2)	0.0578 (18)	0.0370 (15)	0.0294 (17)	−0.0215 (16)	−0.0194 (14)
C5	0.065 (2)	0.083 (2)	0.0399 (16)	0.0015 (17)	−0.0088 (15)	−0.0293 (16)
C6	0.0531 (18)	0.0654 (18)	0.0510 (16)	−0.0007 (14)	−0.0160 (14)	−0.0306 (15)
C7	0.078 (2)	0.082 (2)	0.075 (2)	0.043 (2)	−0.050 (2)	−0.053 (2)
C8	0.0376 (15)	0.0638 (18)	0.0451 (15)	−0.0141 (13)	−0.0039 (12)	−0.0053 (13)
C9	0.0418 (15)	0.0581 (17)	0.0529 (16)	−0.0030 (13)	−0.0104 (13)	−0.0222 (14)
C10	0.0482 (18)	0.082 (2)	0.087 (3)	0.0086 (16)	−0.0295 (18)	−0.047 (2)
C11	0.0572 (17)	0.0461 (15)	0.0410 (14)	−0.0214 (13)	0.0012 (13)	−0.0186 (12)
C12	0.0381 (15)	0.0631 (17)	0.0495 (16)	−0.0100 (13)	−0.0024 (13)	−0.0237 (14)
C13	0.0550 (17)	0.0365 (13)	0.0316 (13)	0.0073 (12)	−0.0056 (12)	−0.0125 (11)
C14	0.0412 (15)	0.0540 (16)	0.0412 (14)	−0.0205 (12)	0.0042 (12)	−0.0209 (12)
C15	0.0368 (14)	0.0440 (14)	0.0427 (14)	−0.0005 (11)	−0.0105 (11)	−0.0196 (11)
C16	0.0402 (15)	0.0434 (14)	0.0347 (13)	−0.0129 (11)	−0.0036 (11)	−0.0053 (11)
C17	0.0384 (14)	0.0367 (13)	0.0330 (12)	0.0015 (10)	−0.0064 (11)	−0.0143 (10)
C18	0.0385 (14)	0.0339 (12)	0.0277 (12)	−0.0056 (10)	−0.0062 (11)	−0.0102 (10)
C19	0.0469 (15)	0.0379 (13)	0.0315 (12)	0.0023 (11)	−0.0077 (11)	−0.0135 (10)
C20	0.0304 (12)	0.0365 (12)	0.0338 (12)	0.0024 (10)	−0.0063 (10)	−0.0144 (10)
C21	0.0297 (13)	0.0474 (14)	0.0388 (14)	−0.0090 (11)	−0.0015 (11)	−0.0094 (11)
C22	0.0332 (13)	0.0389 (13)	0.0284 (11)	−0.0043 (10)	−0.0040 (10)	−0.0127 (10)
Cl1	0.0465 (4)	0.0581 (4)	0.0330 (3)	−0.0189 (3)	0.0043 (3)	−0.0112 (3)
Cl2	0.0389 (3)	0.0525 (4)	0.0368 (3)	−0.0112 (3)	−0.0077 (3)	−0.0147 (3)
N1	0.0725 (17)	0.0467 (13)	0.0350 (12)	−0.0167 (12)	0.0062 (11)	−0.0091 (10)
N2	0.0482 (14)	0.0619 (15)	0.0474 (13)	−0.0193 (11)	−0.0101 (11)	−0.0148 (12)
N3	0.0565 (14)	0.0484 (12)	0.0400 (12)	−0.0222 (11)	0.0037 (11)	−0.0141 (10)
N4	0.0472 (13)	0.0543 (13)	0.0394 (12)	−0.0158 (11)	−0.0071 (10)	−0.0096 (10)
N5	0.0417 (12)	0.0358 (10)	0.0270 (10)	−0.0091 (9)	−0.0025 (9)	−0.0102 (8)
N6	0.0319 (11)	0.0385 (11)	0.0296 (10)	−0.0057 (8)	−0.0031 (9)	−0.0089 (8)
N7	0.0415 (12)	0.0348 (10)	0.0263 (9)	−0.0062 (9)	−0.0036 (9)	−0.0093 (8)
N8	0.0340 (11)	0.0387 (11)	0.0365 (11)	−0.0030 (9)	−0.0090 (9)	−0.0133 (9)
N9	0.0363 (11)	0.0347 (10)	0.0320 (10)	−0.0029 (8)	−0.0087 (9)	−0.0106 (8)
N10	0.0364 (11)	0.0398 (11)	0.0309 (10)	−0.0097 (8)	−0.0014 (9)	−0.0145 (9)

### Geometric parameters (Å, º)

**Table:**

Zn1—N6	2.012 (2)	C10—H10	0.9300
Zn1—N10	2.035 (2)	C11—C14	1.345 (4)
Zn1—Cl2	2.2413 (8)	C11—N7	1.370 (3)
Zn1—Cl1	2.2431 (12)	C11—H11	0.9300
C1—C18	1.482 (3)	C12—C20	1.383 (4)
C1—H1A	0.9600	C12—H12	0.9300
C1—H1B	0.9600	C13—N1	1.376 (3)
C1—H1C	0.9600	C13—C17	1.390 (3)
C2—N8	1.449 (3)	C14—N10	1.385 (3)
C2—N9	1.457 (3)	C14—H14	0.9300
C2—H2A	0.9700	C15—N2	1.370 (3)
C2—H2B	0.9700	C15—C20	1.388 (3)
C3—C12	1.381 (4)	C16—C21	1.335 (3)
C3—C5	1.399 (4)	C16—N9	1.374 (3)
C3—H3	0.9300	C16—H16	0.9300
C4—C7	1.358 (5)	C17—N5	1.361 (3)
C4—C13	1.404 (4)	C18—N10	1.319 (3)
C4—H4	0.9300	C18—N7	1.353 (3)
C5—C6	1.353 (4)	C19—N5	1.441 (3)
C5—H5	0.9300	C19—N7	1.458 (3)
C6—C15	1.401 (4)	C19—H19A	0.9700
C6—H6	0.9300	C19—H19B	0.9700
C7—C10	1.405 (5)	C20—N8	1.369 (3)
C7—H7	0.9300	C21—N6	1.383 (3)
C8—C22	1.477 (3)	C21—H21	0.9300
C8—H8A	0.9600	C22—N6	1.329 (3)
C8—H8B	0.9600	C22—N9	1.353 (3)
C8—H8C	0.9600	N1—N3	1.294 (3)
C9—C10	1.359 (4)	N2—N4	1.296 (3)
C9—C17	1.387 (4)	N3—N5	1.360 (3)
C9—H9	0.9300	N4—N8	1.367 (3)
			
N6—Zn1—N10	106.75 (8)	C17—C13—C4	120.2 (3)
N6—Zn1—Cl2	106.36 (6)	C11—C14—N10	109.3 (2)
N10—Zn1—Cl2	110.35 (6)	C11—C14—H14	125.4
N6—Zn1—Cl1	116.35 (6)	N10—C14—H14	125.4
N10—Zn1—Cl1	103.11 (6)	N2—C15—C20	109.1 (2)
Cl2—Zn1—Cl1	113.66 (3)	N2—C15—C6	130.3 (2)
C18—C1—H1A	109.5	C20—C15—C6	120.6 (2)
C18—C1—H1B	109.5	C21—C16—N9	106.3 (2)
H1A—C1—H1B	109.5	C21—C16—H16	126.9
C18—C1—H1C	109.5	N9—C16—H16	126.9
H1A—C1—H1C	109.5	N5—C17—C13	104.0 (2)
H1B—C1—H1C	109.5	N5—C17—C9	133.1 (2)
N8—C2—N9	114.16 (19)	C13—C17—C9	122.8 (2)
N8—C2—H2A	108.7	N10—C18—N7	110.1 (2)
N9—C2—H2A	108.7	N10—C18—C1	126.2 (2)
N8—C2—H2B	108.7	N7—C18—C1	123.7 (2)
N9—C2—H2B	108.7	N5—C19—N7	112.75 (19)
H2A—C2—H2B	107.6	N5—C19—H19A	109.0
C12—C3—C5	122.4 (3)	N7—C19—H19A	109.0
C12—C3—H3	118.8	N5—C19—H19B	109.0
C5—C3—H3	118.8	N7—C19—H19B	109.0
C7—C4—C13	117.1 (3)	H19A—C19—H19B	107.8
C7—C4—H4	121.5	N8—C20—C12	133.3 (2)
C13—C4—H4	121.5	N8—C20—C15	103.8 (2)
C6—C5—C3	121.8 (3)	C12—C20—C15	122.8 (2)
C6—C5—H5	119.1	C16—C21—N6	109.7 (2)
C3—C5—H5	119.1	C16—C21—H21	125.2
C5—C6—C15	117.0 (3)	N6—C21—H21	125.2
C5—C6—H6	121.5	N6—C22—N9	109.2 (2)
C15—C6—H6	121.5	N6—C22—C8	126.3 (2)
C4—C7—C10	121.6 (3)	N9—C22—C8	124.6 (2)
C4—C7—H7	119.2	N3—N1—C13	108.5 (2)
C10—C7—H7	119.2	N4—N2—C15	108.6 (2)
C22—C8—H8A	109.5	N1—N3—N5	108.8 (2)
C22—C8—H8B	109.5	N2—N4—N8	108.6 (2)
H8A—C8—H8B	109.5	C17—N5—N3	110.18 (19)
C22—C8—H8C	109.5	C17—N5—C19	129.7 (2)
H8A—C8—H8C	109.5	N3—N5—C19	119.62 (19)
H8B—C8—H8C	109.5	C22—N6—C21	106.54 (19)
C10—C9—C17	115.8 (3)	C22—N6—Zn1	130.58 (16)
C10—C9—H9	122.1	C21—N6—Zn1	122.47 (15)
C17—C9—H9	122.1	C18—N7—C11	107.9 (2)
C9—C10—C7	122.5 (3)	C18—N7—C19	126.8 (2)
C9—C10—H10	118.7	C11—N7—C19	125.3 (2)
C7—C10—H10	118.7	N4—N8—C20	109.87 (19)
C14—C11—N7	106.4 (2)	N4—N8—C2	118.9 (2)
C14—C11—H11	126.8	C20—N8—C2	129.8 (2)
N7—C11—H11	126.8	C22—N9—C16	108.33 (19)
C20—C12—C3	115.4 (3)	C22—N9—C2	126.1 (2)
C20—C12—H12	122.3	C16—N9—C2	125.4 (2)
C3—C12—H12	122.3	C18—N10—C14	106.4 (2)
N1—C13—C17	108.6 (2)	C18—N10—Zn1	129.92 (16)
N1—C13—C4	131.1 (3)	C14—N10—Zn1	123.69 (17)
			
C12—C3—C5—C6	0.6 (5)	C16—C21—N6—Zn1	−172.67 (17)
C3—C5—C6—C15	−1.1 (4)	N10—Zn1—N6—C22	−91.4 (2)
C13—C4—C7—C10	−1.0 (4)	Cl2—Zn1—N6—C22	150.73 (19)
C17—C9—C10—C7	−0.5 (4)	Cl1—Zn1—N6—C22	23.0 (2)
C4—C7—C10—C9	1.4 (5)	N10—Zn1—N6—C21	80.14 (19)
C5—C3—C12—C20	0.4 (4)	Cl2—Zn1—N6—C21	−37.69 (19)
C7—C4—C13—N1	−176.0 (3)	Cl1—Zn1—N6—C21	−165.44 (16)
C7—C4—C13—C17	−0.1 (4)	N10—C18—N7—C11	0.2 (3)
N7—C11—C14—N10	−0.3 (3)	C1—C18—N7—C11	179.7 (2)
C5—C6—C15—N2	−176.4 (3)	N10—C18—N7—C19	178.32 (19)
C5—C6—C15—C20	0.7 (4)	C1—C18—N7—C19	−2.1 (4)
N1—C13—C17—N5	0.3 (3)	C14—C11—N7—C18	0.1 (3)
C4—C13—C17—N5	−176.4 (2)	C14—C11—N7—C19	−178.1 (2)
N1—C13—C17—C9	177.8 (2)	N5—C19—N7—C18	77.5 (3)
C4—C13—C17—C9	1.0 (4)	N5—C19—N7—C11	−104.7 (3)
C10—C9—C17—N5	175.9 (3)	N2—N4—N8—C20	−1.5 (3)
C10—C9—C17—C13	−0.7 (4)	N2—N4—N8—C2	−169.2 (2)
C3—C12—C20—N8	176.7 (3)	C12—C20—N8—N4	−176.8 (3)
C3—C12—C20—C15	−0.8 (4)	C15—C20—N8—N4	1.0 (3)
N2—C15—C20—N8	−0.1 (3)	C12—C20—N8—C2	−10.9 (4)
C6—C15—C20—N8	−177.8 (2)	C15—C20—N8—C2	166.9 (2)
N2—C15—C20—C12	178.0 (2)	N9—C2—N8—N4	−96.1 (3)
C6—C15—C20—C12	0.3 (4)	N9—C2—N8—C20	99.1 (3)
N9—C16—C21—N6	−0.4 (3)	N6—C22—N9—C16	0.4 (3)
C17—C13—N1—N3	−0.5 (3)	C8—C22—N9—C16	−179.2 (2)
C4—C13—N1—N3	175.8 (3)	N6—C22—N9—C2	176.3 (2)
C20—C15—N2—N4	−0.8 (3)	C8—C22—N9—C2	−3.3 (4)
C6—C15—N2—N4	176.5 (3)	C21—C16—N9—C22	0.1 (3)
C13—N1—N3—N5	0.5 (3)	C21—C16—N9—C2	−175.9 (2)
C15—N2—N4—N8	1.4 (3)	N8—C2—N9—C22	82.1 (3)
C13—C17—N5—N3	0.0 (3)	N8—C2—N9—C16	−102.7 (3)
C9—C17—N5—N3	−177.1 (3)	N7—C18—N10—C14	−0.4 (3)
C13—C17—N5—C19	171.4 (2)	C1—C18—N10—C14	−179.9 (2)
C9—C17—N5—C19	−5.7 (4)	N7—C18—N10—Zn1	−179.89 (14)
N1—N3—N5—C17	−0.3 (3)	C1—C18—N10—Zn1	0.6 (3)
N1—N3—N5—C19	−172.7 (2)	C11—C14—N10—C18	0.4 (3)
N7—C19—N5—C17	79.1 (3)	C11—C14—N10—Zn1	179.98 (17)
N7—C19—N5—N3	−110.1 (2)	N6—Zn1—N10—C18	−69.1 (2)
N9—C22—N6—C21	−0.6 (3)	Cl2—Zn1—N10—C18	46.1 (2)
C8—C22—N6—C21	178.9 (2)	Cl1—Zn1—N10—C18	167.84 (18)
N9—C22—N6—Zn1	171.97 (15)	N6—Zn1—N10—C14	111.50 (19)
C8—C22—N6—Zn1	−8.5 (4)	Cl2—Zn1—N10—C14	−133.32 (18)
C16—C21—N6—C22	0.7 (3)	Cl1—Zn1—N10—C14	−11.60 (19)
